# Editorial: Drug discovery for leishmaniasis and chagas disease: compound design and therapeutic strategies

**DOI:** 10.3389/fphar.2025.1718438

**Published:** 2025-10-14

**Authors:** Karunakaran Kalesh, Euzébio Guimarães Barbosa, Alessandro Kappel Jordão

**Affiliations:** ^1^ School of Health and Life Sciences, Teesside University, Middlesbrough, United Kingdom; ^2^ Pharmacy Department, Federal University of Rio Grande do Norte, Natal, Brazil

**Keywords:** leishmaniasis, neglected tropical diseases, antileishmanial drug discovery, N-myristoyltransferase (NMT) inhibitors, natural products, quinoline-4-carboxylic acids, host-directed therapy, iron oxide nanoparticles

## 1 Why this Research Topic matters

Neglected tropical diseases (NTDs) such as leishmaniasis and Chagas disease remain pressing global-health challenges. Caused by the protozoa *Leishmania* and *Trypanosoma cruzi*, these infections persist where poverty, poor access to care and vector exposure intersect, and their global impact is growing with migration and climate change (de Brito et al., 2024). Although therapeutic options have improved, limitations persist, such as long regimens, toxicity, variable efficacy, and the threat of resistance.

This Research Topic was designed to showcase strategies that could change therapeutic trajectories, moving beyond incrementalism. These approaches include rational compound design anchored in target biology and pharmacokinetics/pharmacodynamics (PK/PD), phenotypic discovery linked to early target deconvolution, host-directed and delivery innovations, and, where appropriate, resistance-aware combinations. Five articles were accepted; all focused on *Leishmania* biology and therapy. While no Chagas disease studies were included, several mechanistic and translational insights are likely applicable to *T. cruzi*, particularly where pathogen pathways or host responses are conserved.

## 2 Key insights and field trajectories

The articles in this Research Topic collectively map a coherent arc for antileishmanial drug discovery: from how we prioritise hits and leads to how we think about pharmacology, resistance, and the host response. Taken together, they delineate common scientific threads with direct implications for the next wave of candidates.

A first theme is the re-emergence of N-myristoyltransferase (NMT) as a compelling antileishmanial target, approached from distinct starting points. Boudou et al. employed the pharmacopeia classic - *Artemisia absinthium* - and show how careful phytochemical work, coupled with structure-guided docking and molecular dynamics, can surface tractable flavonoids (e.g., apigenin) with predicted NMT engagement and reasonable drug-likeness. In parallel, da Silva et al. explore 2-aryl-quinoline-4-carboxylic acid derivatives and identify NMT as the target through an inverse virtual screening and docking workflow, again reinforced by MD stability and favourable *in silico* ADMET. When different discovery routes independently point to the same essential enzyme, it boosts confidence in its relevance and clarifies the medicinal-chemistry path to more selective and potent inhibitors. What is now required are decisive whole-cell data in intracellular amastigotes, direct target engagement (e.g., resistance selection plus whole-genome sequencing, chemoproteomics, CETSA/thermal profiling), and early liabilities mapping to avoid class-wide pitfalls.

Natural products remain a productive source of chemically privileged scaffolds, provided we apply a developability lens from the outset. Marques et al. used bioassay-guided isolation of cucurbitacin-type triterpenoids from *Momordica charantia* and reported moderate activity against both promastigotes and intracellular amastigotes with encouraging selectivity in primary macrophages. Triterpenoids carry well-known potency-toxicity trade-offs; nevertheless, their modular stereochemistry and handle-rich cores invite structure-activity work to improve solubility, permeability, and microsomal stability while testing whether efficacy can be uncoupled from cytotoxicity. Attention to tissue distribution is equally important: for VL, compounds must reach macrophage-dense spleen and liver; for CL, achieving sustained lesion-site exposure may allow dose-sparing local therapy.

Two authoritative reviews widen the aperture from parasite-centric chemistry to host-directed and immunological strategies. Palomino-Cano et al. synthesise a growing literature on macrophage polarisation and the iron tug-of-war in *Leishmania* infection and argue for the use of iron oxide nanoparticles (IONPs) as an immunotherapeutic lever. The review synthesises evidence that iron metabolism is a fulcrum in *Leishmania* infection and posits IONPs as an immunotherapeutic lever to shift M2-like, permissive macrophages toward an M1-like, leishmanicidal state. The appeal is twofold: lowering selective pressure for parasite resistance and using innate phagocytic uptake for targeted delivery. But translation will depend on getting the dose right, ensuring the nanoparticles go where they should, and proving safety because immune activation in visceral organs is a double-edged sword. Pairing such host-directed tools with vetted antileishmanial doublets, and matching pharmacokinetics across components could, in-principle, yield shorter, safer regimens.

Complementing this, Tiwari et al. explain why durable protection against leishmaniasis is hard and how we might engineer it. Lasting protection depends on Th1-biased CD4^+^ T cells and strong central/effector/tissue-resident memory, but maintaining this protection often requires persistent antigen or periodic re-exposure. This has practical implications for drug development and vaccination: sterilising cures may not always be immunologically optimal, and vaccine candidates must be formulated and targeted to generate tissue-appropriate memory without undue reactogenicity. As long-acting formulations and combination therapies advance, co-measuring immunological endpoints in preclinical and clinical studies could uncover synergies or reveal unintended trade-offs we should address early.

Taken together, the Research Topic argues for integrated, PK/PD-aware discovery that links target choice to exposure at disease-relevant sites, uses early resistance-risk assessment to guide combinations, and embraces host-directed adjuncts when they can improve efficacy or shorten therapy. Although this Research Topic received no original Chagas disease studies, the mechanistic threads identified here, namely, NMT inhibition, triterpenoid scaffolds, macrophage/iron axis manipulation, and rational immuno-pharmacology are readily testable in *T. cruzi*, with appropriate attention to cardiac tissue penetration and chronic-phase biology. The field is well placed to convert these insights into candidates that are not only potent *in vitro* but practical, safe, and durable where they are most needed.

## 3 New frontiers to watch

A notable shift in the antileishmanial drug discovery is the maturation of truly novel mechanisms into patient studies. The first-in-class *Leishmania* proteasome inhibitor LXE408 has advanced to Phase II trials for both visceral and cutaneous leishmaniasis (DNDi Press Release, 2024). In parallel, clinical-stage inhibitors of cyclin-dependent-related kinase 12 (CRK12) exemplify a second orthogonal pathway, born from phenotypic hits but now supported by target annotation and preclinical efficacy (Wyllie et al., 2018). Finally, oxaboroles that inhibit the mRNA-processing endonuclease CPSF3 (e.g., DNDi-6148 and next-gen analogues) broaden the portfolio with a chemically distinct class capable of potent whole-cell activity (Mowbray et al., 2021). Together, these programmes mark a diversification beyond legacy chemotypes, with a clear emphasis on oral, short-course regimens.

An emerging line of evidence highlights the impact of the parasite virome, particularly *Leishmania* RNA virus (LRV1/LRV2), on disease severity and treatment outcomes. Meta-analytic and regional studies report substantial LRV prevalence across *Leishmania* species, and link viral carriage to heightened inflammation, mucocutaneous disease propensity, and risk of therapeutic failure (Yektaeian et al., 2025). For developers, this raises practical questions: should trials stratify by LRV status? Could antiviral or TLR-pathway modulators serve as adjuncts in LRV-positive disease? Incorporating virome diagnostics and pre-specified subgroup analyses may reduce noise in efficacy signals and point to regimen tailoring where it matters.

Beyond molecules, how we deliver them is changing. Intralesional and other locoregional approaches (including liposomal/depot formulations) are showing potential to reduce systemic exposure while achieving high lesion-site concentrations, particularly relevant for cutaneous leishmaniasis, where field-friendly regimens are needed. In parallel, long-acting strategies and nano-enabled carriers aim to match pharmacokinetics to the slow-cycling intracellular niche, shorten dosing schedules, and improve adherence. A complementary insight from recent human studies is that clinical cure does not always equal sterilising cure; parasite persistence and host biosignatures of failure argue for regimens that combine potent debulking with immune-compatible delivery, and for trials that co-measure exposure at target tissues alongside immune endpoints.

Although no articles within this Research Topic focused on Chagas disease, it is noteworthy that the search for alternative treatments is an area of constant research. A key example is the DNDi-supported BENDITA trial conducted by Torrico et al. (2021), which evaluated new regimens of benznidazole. The study concluded that benznidazole induced an effective antiparasitic response in adult patients with chronic Chagas disease, regardless of treatment duration, dose, or combination with fosravuconazole. Furthermore, shorter or reduced-dose regimens were well tolerated and could substantially improve treatment tolerability and accessibility. However, the authors emphasize that further studies are needed to confirm these results.

## 4 Outlook: toward better, shorter safer regimens

The near-term opportunity is to convert converging biology into practical regimens. On the molecule side, programmes that have broken new mechanistic ground - parasite proteasome, CRK12, CPSF3 -should prioritise differentiation on safety and duration, not just potency. That means early and repeated links between whole-cell activity and exposure at the relevant tissues, resistance-risk mapping to inform combinations, and formulation work that anticipates use in resource-limited settings.

On the trial design side, one upgrade would add real value: build immune-PK/PD co-readouts into studies. Alongside clinical endpoints, measure drug levels in relevant tissues (e.g., dermal microdialysis or lesional aspirates where feasible) and track immune markers linked to durable control. This reduces noise, clarifies why regimens succeed or fail, and guides smarter combination choices.

On the delivery side, field-friendly approaches can shorten courses and improve tolerability. Intralesional or depot strategies for cutaneous disease, long-acting or nanoparticle-enabled options for visceral disease, and targeted macrophage delivery are all promising - provided dose, biodistribution, and safety are established with the same rigour applied to small molecules. Where host-directed adjuncts are considered, regimens should be designed to boost macrophage-mediated killing without collateral tissue injury, with conservative stopping rules and immune monitoring built in.

Finally, while this Research Topic focuses on *Leishmania*, several principles translate to Chagas disease: diversify targets with early genetic/chemical validation; design for cardiac exposure and chronic-phase pharmacology; and plan combinations that pair orthogonal mechanisms with matched kinetics. If the field embraces these practices, we stand to deliver shorter, safer, and more durable therapies that are usable where they are most needed.
